# Outcomes of patients with a non-diagnostic initial bronchoscopy for suspected thoracic malignancy

**DOI:** 10.1097/MD.0000000000022772

**Published:** 2020-10-23

**Authors:** Maneesh Gaddam, Stephen Paff, Sindhaghatta Venkatram, Gilda Diaz-Fuentes

**Affiliations:** a Pulmonary Fellow, Division of Pulmonary and Critical Care Medicine; b Data Scientist, Department of Internal Medicine; c Associate Professor of Clinical Medicine, Division of Pulmonary and Critical Care Medicine, BronxCareHealth System, 1650 Grand Concourse, Bronx, NY 10457, Affiliated with Icahn School of Medicine at Mount Sinai.

**Keywords:** bronchoscopy, endobronchial biopsy, endobronchial ultrasound, non-diagnostic, transbronchial biopsy, yield

## Abstract

Lung cancer is 1 of the leading causes of cancer-related deaths and bronchoscopy is an essential tool for the diagnosis. The diagnostic yield varies based on the characteristics of the lesion and bronchoscopic techniques employed. There is limited data regarding outcomes of patients suspected of thoracic malignancies with a non-diagnostic initial bronchoscopy. The goal of the study was to evaluate the outcomes of patients with a non-diagnostic bronchoscopy for suspected thoracic malignancies and to evaluate variables predictive of a diagnostic bronchoscopy.

Retrospective analysis of adult patients at BronxCare Hospital Center who underwent bronchoscopy for suspected thoracic malignancy. The study period was January 2012 to February 2019. Exclusion criteria included patients who underwent only inspection bronchoscopy or bronchoalveolar lavage as the diagnostic yield for malignancy with these techniques is low. All other bronchoscopic procedures were included that is, endobronchial biopsies, transbronchial biopsies, and endobronchial ultrasound guided-transbronchial needle aspiration. Bronchoscopy was considered diagnostic when a specific histopathological diagnosis was established.

311 patients underwent bronchoscopy to rule out malignancy. A diagnosis was obtained in 153 (49.2%) patients, 81 (52.9%) had primary lung cancer and 14 (9.15%) other malignancies. 158 (50.8%) patients had initial non-diagnostic bronchoscopy; 86 (54.43%) were lost to follow up. Of the remaining 72 (45.57%) patients, radiological resolution or stability was observed in 51 (70.8%) patients. Primary lung cancer was found in 13 (18.05%) patients and other malignancies in 5 (6.94%). Predictive of a diagnostic bronchoscopy was the performance of endobronchial biopsies and endobronchial ultrasound guided-transbronchial needle aspiration.

This study highlights some of the barriers to the timely diagnosis of thoracic malignancies. Following patients with a non-diagnostic procedure as well as all those patients with diagnosed malignancies it of the utmost importance. In patients available for follow up, close to 25% of additional cases with treatable malignancy could be identified and patients diagnosed with cancer could receive timely treatment.

## 1 Introduction

Lung cancer, despite many medical advances, remains as the leading cause of cancer-related deaths worldwide. Flexible fiberoptic bronchoscopy (FFB) plays an essential role in the diagnosis of lung cancer and other thoracic malignancies. The diagnostic yield of FFB varies significantly based on the characteristic and location of the lesion and the bronchoscopic techniques employed.^[1–5]^ There is sparse data regarding the outcome or follow up of patients suspected of thoracic malignancies who had a non-diagnostic initial bronchoscopy.^[6–9]^

The goal of this study was to evaluate the outcomes of patients with an initial non-diagnostic bronchoscopy for suspected thoracic malignancies. In addition, we evaluated variables predictive of a diagnostic bronchoscopy.

## 2 Materials and methods

### 2.1 Study design and patients

This was a retrospective cohort study conducted at BronxCare Hospital Center which is a 972 bedded inner-city community teaching hospital serving the South and Central Bronx. All adult patients who underwent FFB during the period of January 2012 to February 2019 for suspected malignancy were included.

Ethics approval: This study protocol adhered to the amended Declaration of Helsinki and was approved by our Institutional Review Board (approval number 10111806).

Exclusion criteria included those patients who underwent only inspection bronchoscopy or bronchoalveolar lavage, as the diagnostic yield for malignancy with these techniques is very low. All others bronchoscopic procedures were included ie endobronchial biopsies (EBBX), transbronchial biopsies (TBBX) and endobronchial ultrasound guided-transbronchial needle aspiration (EBUS-TBNA).

All bronchoscopies were performed using a standard flexible bronchoscopy (Olympus America Inc; Melville, NY). Based on the pre-procedural risk evaluation, procedures were either done under local anesthesia with conscious sedation in the bronchoscopy suite or under general anesthesia in the operating room. All TBBXs were performed under fluoroscopic guidance. All EBUS-TBNA were performed in the operating room under general anesthesia.

### 2.2 Data abstraction

All data including demographic, clinical information and bronchoscopic procedures were retrospectively extracted from medical records. Radiological findings were obtained from radiology reports of the thoracic imaging.

### 2.3 Definition of diagnostic and non-diagnostic bronchoscopy

A bronchoscopy was considered diagnostic when a histopathological diagnosis was established with the procedure. All other bronchoscopies including those with no established histopathological diagnosis and those patients needing follow-up imaging or follow-up procedures were considered non-diagnostic.

### 2.4 Statistical analysis

We identified 311 patients who had bronchoscopy for suspected malignancy. To assess the relationship between each independent variable and their diagnosis, chi-squared were used for categorical independent variables, and *t*-tests for quantitative independent variables.

Two logistic regression models were used to assess the predictive importance of key symptoms and demographic variables. The first logistic regression model analyzed the role of the variables, TBBX, EBBX, and EBUS-TBNA, on diagnosis, and the second analyzed the role of the variables, gender, HIV status, fever, weight loss, hemoptysis, chest roentgenogram, bronchoalveolar lavage Cytology, and bronchoscopy findings.

The likeliness-ratio test determines whether the given variable has a statistically significant role by assessing whether excluding the given variable from the logistical regression model has a statistically significant impact on the model performance. If the difference is statistically significant, then the original model including that variable fits the data significantly better, demonstrating that that variable has an important impact on the logistic regression model in determining a diagnosis or non-diagnosis. The B coefficient represents each variable's unstandardized regression coefficient within the logistic regression model. A positive coefficient indicates a direct relationship in diagnosis and negative an indirect relationship. Exp(B) represents the odds-ratio, measuring the strength of that variable's association with a diagnosis. The exp(B) would be the expected change in the output variable for a 1-unit increase in the given input variable.

An alpha of 0.05 was the threshold for all of these tests. Categorical variables were expressed as counts (percentage), while continuous variables were expressed as means ± standard deviations (SD). The statistical analysis was carried out using SPSS 20.0 and Jupyter Notebook.

## 3 Results

### 3.1 Outcomes of the initial bronchoscopy

**Figure 1:**
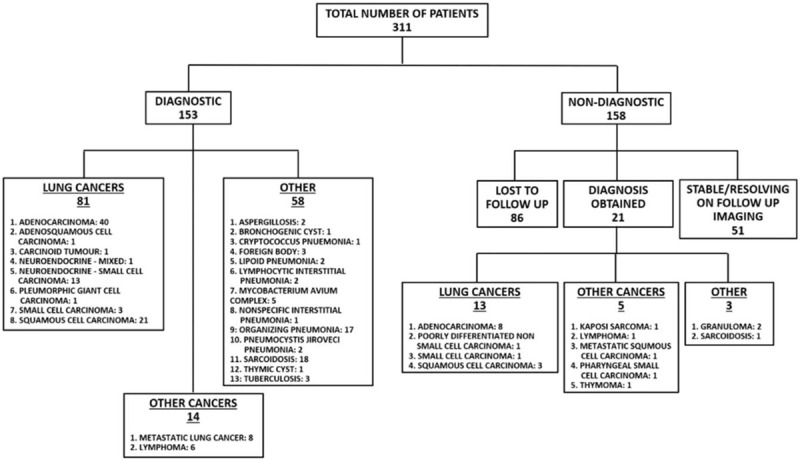
Flow chart showing outcomes of initial bronchoscopy.

A total of 311 patients underwent bronchoscopy to rule out malignancy. A diagnosis was obtained in 153 (49.2%) of the patients; 81 (52.9%) had primary lung cancers and 14 (9.15%) other malignancies including metastatic lung cancers and lymphomas. Other diagnoses were identified in 58 (37.91%) patients. (Fig. 1). Of the 95 patients identified to have thoracic malignancies, 15 (15.8%) were lost to follow up; all of them were aware of the diagnosis.

A total of 158 (50.8%) patients had an initial non-diagnostic FFB; 86 (54.43%) of those patients were lost to follow up. The remaining 72 (45.57%) patients were followed for at least 6 months and they required follow up imaging or further procedures to reach a final diagnosis. Radiological resolution or stability was observed in 51 (70.8%) of the 72 patients available for follow up; primary lung cancers were found in 13 (18.05%) and other thoracic malignancies in 5 (6.94%) patients. Other diagnoses were found in 3 (4.17%) patients and included granuloma and sarcoidosis.

Various methods were required to establish a final diagnosis on those patients with an initial non-diagnostic bronchoscopy. There were 18 additional malignancies identified on further intervention; in 7 patients, the diagnoses were obtained by CT guided biopsies, 5 needed lung wedge resection, 3 lobectomy and 3 mediastinoscopy. One lung granuloma was identified on lobectomy, the other after wedge resection. Mediastinoscopy yielded the diagnosis of sarcoidosis.

### 3.2 Comparison of diagnostic and non-diagnostic bronchoscopy

A comparison of demographics, comorbidities and symptoms at presentation between the diagnostic and non-diagnostic groups is shown in Table 1. There were no differences in the groups regarding basics demographics and comorbid conditions including smoking history, HIV status, obstructive airway disease and history of malignancy. The most common symptoms at presentation in both groups were cough and shortness of breath.

**Table 1:**
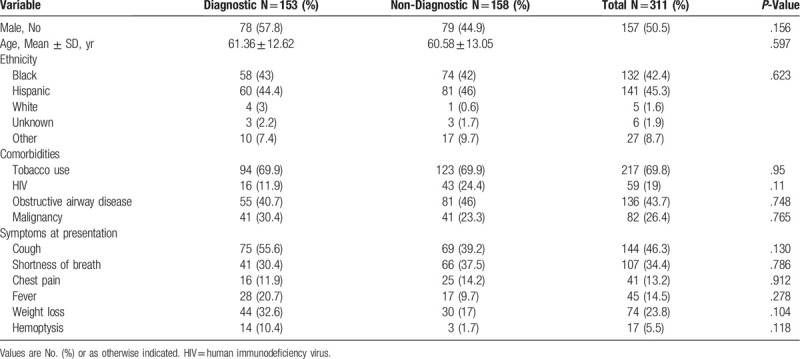
Demographics, comorbidities, and symptoms at presentation.

The most common radiological findings on chest roentgenogram were unilateral or bilateral infiltrates followed by mass. No differences between the groups were seen. Table 2

**Table 2:**
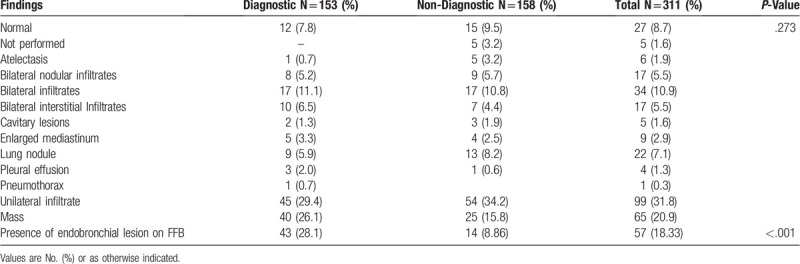
Chest imaging and bronchoscopic findings.

There were 3 diagnostic procedures performed in the patients, TBBX, EBBX, and or EBUS-TBNA. One hundred forty three (46.9%) patients underwent EBUS-TBNA, 248 patients (79.74%) had TBBX and 99 patients (31.83%) had EBBX in the cohort. Patients in the diagnostic group had significantly higher numbers of TBBX performed, but in logistic regression analysis, the bronchoscopic procedures with the higher diagnostic yield were EBBX and EBUS-TBNA. (Tables 3 and 4)

**Table 3:**
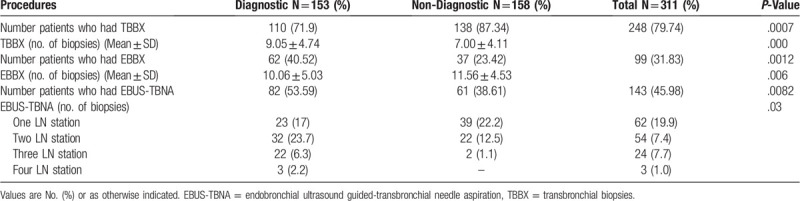
Bronchoscopic Procedures.

**Table 4:**
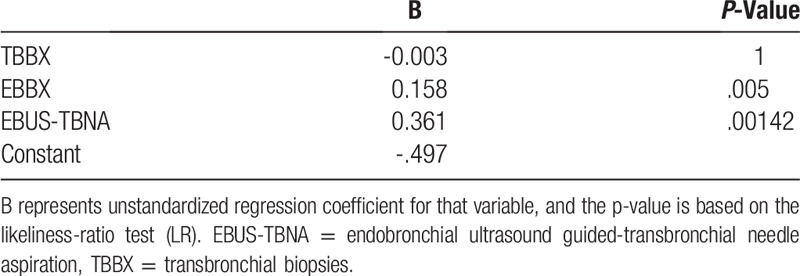
Logistic regression model #1.

The second logistic regression model (Table 5) assessed the predictive importance of key symptomatic variables, imaging and bronchoscopic findings including sex, HIV, fever, weight loss, hemoptysis, chest roentgenogram, and bronchoscopy findings of endobronchial lesions. While symptoms and imaging findings did not have a positive impact on diagnosis, the presence of an endobronchial lesion (B = 0.300874, *P* = .000^∗^) appears to have a significant, positive impact on whether there is a diagnosis.

**Table 5:**
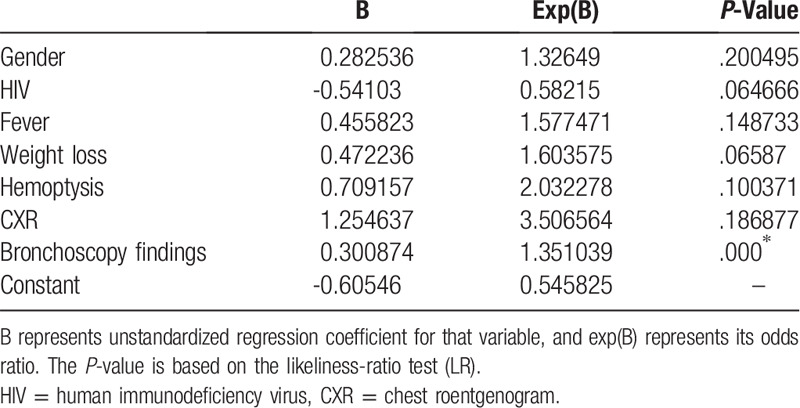
Logistic regression model #2.

Bronchoscopy related complications were seen in 56 (18%) patients, bleeding in 47 (15.1%) which resolved with the use of topical epinephrine and/or cold saline, respiratory failure requiring transient use of mechanical ventilation in 6 (1.9%), pneumothorax in 2(0.6%) and arrhythmia in 1 (0.3%) patients.

## 4 Discussion

The diagnostic bronchoscopic yield of 49.2% for suspected thoracic malignancy at our institution was similar to other studies.^[6,7,10,11]^ Consistent with other reports, the diagnostic yield was higher in the presence of endobronchial lesions or performance of EBUS-TBNA ^[8,12,13,14,15]^ Our study did not identify any demographic, clinical factors or radiographic findings predictive of a diagnostic or non-diagnostic bronchoscopy. The time frame to establish the diagnosis was variable; the main contributing factors included difficulty in contacting and tracing patients, patients missing follow up appointments, time waiting for additional imaging required prior to scheduling further interventions, waiting time to be seen by other specialist and booking of additional procedures.

The overall no show rate for scheduled follow-up appointments at our institution is approximately 40%. Concerning was the findings that 54.4% of our patients with a non-diagnostic bronchoscopy were lost to follow up. This is despite an institutional automatic phone reminder and recall system. In those patients available for follow up, malignancies were identified in 18 (25%) of the patients. The study highlights a major clinical and social concern; the possibility of missing an additional 20 patients with undiagnosed malignancy in the patients who were lost for follow up. The risk of thoracic malignancies in our community is high considering the high prevalence of active cigarette smokers. Another area of concern is that up to 15% of patients with an initial diagnosis of thoracic malignancies were missed for follow up for the treatment of the cancer.

Various factors play a role in the low follow up rates. It is reported that health in the United States is significantly patterned along both socioeconomic and racial lines, suggesting a correlation between the hierarchies of social advantage and health.^[16]^ Given the fact that our institution serves 1 of the poorest congressional districts in the nation, along with the health care disparities are likely responsible for poor follow up rates. The reasons for low follow up rates in an inner-city population are multifactorial.^[17–19]^ Common among many include social support, ethnicity, cultural belief, type of insurance coverage and undocumented status.^[20,21]^

Based on our study findings and in addition to our institutional systems to recall and track patients for follow up, the following additional interventions were implemented to ensure better follow up. Additional contact detail of the patient is obtained when planning for bronchoscopy, a follow up appointment within a week of the procedure is set up in agreement with the patient, a reminder call by clinic staff in addition of automatic hospital calls are done the day prior to appointment. If all of those fail, patients are sent a recall letter and a social and community care worker are involved to locate the patients. Our preliminary, unpublished data shows that the no show rate after bronchoscopies is trending down.

Our study has several strengths. First, a clear definition for a diagnostic and non-diagnostic bronchoscopy was included; second, this is 1 of the very few studies looking at outcomes of patients with non-diagnostic bronchoscopy and a follow up of at least 6 months. This established new insight into the outcomes of these procedures. Third, the study performed at an inner-city setting, providing information regarding limitations to diagnose malignancies in similar communities. Fourth, we examined the relationship between clinical and radiological parameters trying to identify variables predictive of a diagnostic procedure.

Limitations of the study include performance in a single center and the retrospective nature of the study. While multiple attempts to contact patients with an initial non-diagnostic procedure were made, it is possible that some of those patients might have decided to seek further medical care or advice at other facilities. That information and the outcome of those patients was not available in this study.

## 5 Conclusions

In summary, our study provides new insights into the outcomes of patients suspected of thoracic malignancies who had an initial non-diagnostic bronchoscopy in an inner-city area. It highlights some of the barriers to the timely diagnosis and the follow up of patients suspected of thoracic malignancies. It is of the utmost importance to follow those patients as well as all those patients with diagnosed malignancies. Close to 25% of additional patients with treatable malignancy could be identified and patients diagnosed with cancer could receive timely treatment. The present results also confirm previous studies that founds an increased bronchoscopic diagnostic with EBBX and EBUS-TBNA.

Clinicians need to be aware of the strengths and barriers and limitations of their local health care systems for patients’ care. Learning the characteristics of the community been served is essential. Development of a multidisciplinary, institutional and local system to improve adherence to care is recommended.

## Author contributions

GDF is the guarantor of this paper and takes responsibility for the content of the manuscript including the data and analysis. SV contributed to the design, planning, initiation, data collection, data analysis, data interpretation, and writing of the manuscript. MG, SP contributed to data collection, data analysis, data interpretation, and writing and take responsibility for data integrity. All authors had access to the data and the final manuscript for approval before submission.

**Conceptualization**: Gilda Diaz-Fuentes, Sindhaghatta Venkatram, Maneesh Gaddam.

**Data curation**: Maneesh Gaddam.

**Formal analysis**: Sindhaghatta Venkatram, Maneesh Gaddam, Stephen Paff.

**Investigation**: Maneesh Gaddam, Stephen Paff.

**Methodology**: Gilda Diaz-Fuentes, Maneesh Gaddam.

**Supervision**: Gilda Diaz-Fuentes.

**Writing – original draft**: Gilda Diaz-Fuentes, Maneesh Gaddam.

**Writing – review & editing**: Sindhaghatta Venkatram.
